# Supplementary material for: Causal interplay between lactose intolerance and gut microbiota: a combined bidirectional Mendelian randomization and in vivo validation study
Source: Front Nutr. 2026 Jun 1;13:1803337. doi: 10.3389/fnut.2026.1803337 (PMC13265576; doi:10.3389/fnut.2026.1803337)
Supplement: Supplementary file 1 [file Data_Sheet_1.zip › supplementary materials/Forward/leave one out cross validation/ebi-a-GCST90027714.finngen_R12_E4_LACTONAS.pdf]

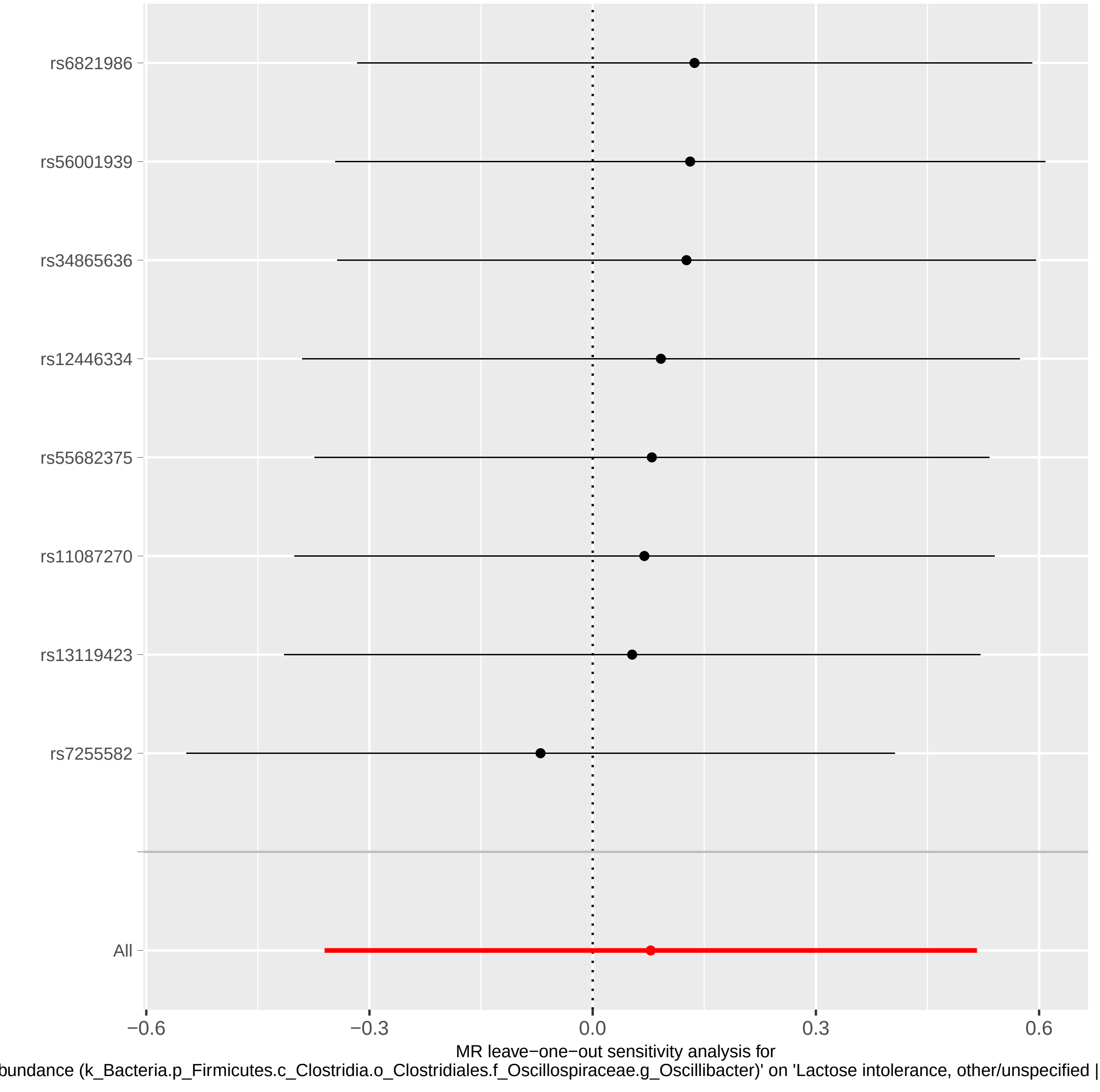
rs6821986
rs56001939
rs34865636
rs12446334
rs55682375
rs11087270
rs13119423
rs7255582
All
−0.6
−0.3
0.0
0.3
0.6
MR leave−one−out sensitivity analysis for
bundance (k_Bacteria.p_Firmicutes.c_Clostridia.o_Clostridiales.f_Oscillospiraceae.g_Oscillibacter)' on 'Lactose intolerance, other/unspecified |
